# Epidemiological, clinical and laboratory features of patients hospitalized with 2009 pandemic influenza in north of Iran

**Published:** 2012

**Authors:** Narges Najafi, Ali Reza Davoudi, Farhang Baba Mahmoodi, Atefe Tayebi, Sharyar Alian, Roya Ghassemian, Ahmad Alikhani

**Affiliations:** 1 Antimicrobial Resistance Research Center, Mazandaran University of Medical Sciences, Sari, Iran.

**Keywords:** Novel influenza A (H1N1), Pandemic, Acute respiratory distress

## Abstract

**Background:**

The clinical manifestations and outcome of influenza infection differ between various patients in the world. The purpose of this study was to assess the clinical manifestations of patients with confirmed or suspected novel H1N1 flu infection in Sari, North of Iran.

**Methods:**

From September 2009 to January 2010, the patients’ data were collected by retrospective chart review of medical records. Laboratory confirmation included a positive RT–PCR (reverse transcriptase–polymerase-chain-reaction assay) from a nasal or pharyngeal swab sample.

**Results:**

Nearly 80% of established patients were in age group of 15-45 years. Approximately 14.6% of female cases were pregnant There was no significant difference in clinical and laboratory characteristics of patients with confirmed H1N1 virus infection to total cases with Influenza Like Illness (ILI). Thirty nine (95.1%) of the established patients had a combination of fever plus sore throat or cough**.** Relative lymphopenia was reported in 36.6%. Pneumonia was the most common complication**.** Acute pericarditis evolved in one case and aseptic meningitis was reported in another.

**Conclusion:**

Precise collecting information of clinical manifestations, risk factors and other characteristics of flu, can help to the early infection detection, timely treatment of patients and proper preventive measurements.

In April 2009, two cases of human infection with novel influenza with swine origin were confirmed by the Center for Disease Control (CDC) for the first time (1). The 2009 H1N1 virus contained a unique combination of gene segments which had not previously been identified in humans or animals (2). In short, many cases of diseases have been reported in the USA, Mexico and the other countries worldwide. Therefore the first pandemic in third thousandth was begun (3). Like the other flu viruses, 2009 H1N1 virus spreads from one person to another through coughing, sneezing, and occasionally through touching objects contaminated with the virus. The incubation period of infection is estimated about 2-7 days. 

The infection usually involves young and middle aged persons. Symptoms include abrupt onset of fever, chills, headache, sore throat, malaise, coryza, non productive cough and occasionally nausea, vomiting and diarrhea. Illness is frequently self limited in one week period, although dry cough or weakness can continue up to 2-4 weeks. Some patients who are more at risk for the complication or death include pregnant women, very obese persons, children smaller than 5 years old or regular aspirin users, and those with immune suppression or chronic underlying disease (4). The most general infection complications related to respiratory system include primary viral pneumonia, secondary bacterial pneumonia, otitis media, acute sinusitis, croup and exacerbation of a chronic lung disease. 

Non respiratory complications which occur uncommonly are encephalopathy, encephalitis, transverse myelitis, guillain barré syndrome, toxic shock syndrome, myositis, myoglobineuria, reys syndrome, myocarditis and pericarditis (4, 5). Collecting information about the clinical and epidemiological aspects of pandemic 2009 H1N1 virus infection is still going on since these characters may be variable in different geographical regions, therefore, regional studies in this subject can provide a better vision of infection .This will help in the case of management or infection prevention if evolve with latter waves of this pandemic. 

## Methods

In a retrospective study of the patients with influenza-like illness (T≥ 38 °C and cough or sore throat) hospitalized in the teaching hospitals of Mazandaran (North of Iran) was conducted. The patients had been admitted in Ghaemshahr Razi Hospital and Sari Imam Khomeini Hospital from September 2009 to January 2010, the data were collected by retrospective chart review of medical records. We took a written consent from all of the patients to use and publish the said medical records. A confirmation RT–PCR (reverse transcriptase polymerase chain reaction assay) from a nasal or pharyngeal swab sample was employed. 

These tests were performed either at. School of Medical Health Laboratory Tehran University of Medical Sciences or the Reference Laboratory of Mazandaran University of Medical Sciences. Finally, the collected data were analyzed using the SPSS version 17 (including descriptive statistics with median values). The differences between the two groups were compared using the chi-square test for the qualitative variables and independent t-test for quantitative variables. The level of significance was at p<0.05. 

Many cases with an influenza-like illness (ILI) had negative RT- PCR test, while these results probably were believed due to the delay or not performing of confirmatory test. Thus, the results were evaluated in two groups (total cases with ILI and confirmed cases of human infection with novel influenza with swine origin).

## Results

In this study, 171 patients were admitted with ILI, but 147 cases were enrolled in this study. Twenty four cases were excluded because of the confirmation of another diagnosis such as, brucellosis, urinary tract infection, infectious mononucleosis, streptococcal pharyngitis and gastroenteritis. Patients with ILI: A total of 147 cases with ILI were hospitalized, 78 (53.1%) were females and 69 (46.9%) were males. The mean of the patient’s age was 32.3±16.7 (range, 5-86) years. Merely 8 (5.4%) patients were 65 years old or older, while 101 (68.7%) were in the age group of 15-45 years. The mean age for the male patients was 32.3±16.4 years, and for the females was 32.3±17.1 years without significant difference between the 2 genders (p=0.98). The most common chief complaint was fever (57.8%). The clinical and laboratory characteristics of the patients are given in table 1. 

The mean time of the beginning of illness to specimen collection was 4.2±3.8 (range 1-30) days. Forty seven patients (31.9.3%) had one or more underlying disease (including asthma, diabetes mellitus, hypertension, COPD, heart failure, chronic renal failure, addiction, obesity, malignancy, transplantation, corticosteroid therapy, chemotherapy). Also, 25 of the 79 female patients were pregnant (table 1). The chest x-ray was performed in 123 patients which was abnormal in 34 (27.6%) cases (unilateral or bilateral infiltration). Almost 16% of patients were leukopenic (WBC<4000), while only 9.5% of cases had leukocytosis (WBC>12000). The most common complication was pneumonia (18 of 147 cases). A total of 84% of patients received antiviral therapy (TAMIFLU®). Five cases were admitted in the ICU and unfortunately 2 patients had expired which both had severe and uncompensated underlying diseases (congestive heart failure and major thallassemia).

**Patients with confirmed novel H1N1 flu infection: **Among the 147 patients with ILI, 41 were positive for H1N1-RT PCR which 22 (53.7%) were males and 19 (46.3%) females. The mean age of these patients was 30.8 ±15.4 (range, 8-79) years. Three (7.3%) patients were older than 65 years and 33 patients (80.5%) were in the age group of 15-45 years. The mean age for the male cases was 29.5±13.9 years and for the females was 32.4±17.3 years with no significant difference in 2 sex groups (p=0.61). 

The mean time from the beginning of illness to sample collection was 0.93±0.47 (range 1-5) days. The mean time where the patients were hospitalized after the beginning of symptoms was 4.2±3.8 days. The most common complaints which patients presented were fever (56.1%), followed by cough (12.2%), headache (9.6%) and dyspnea (9.6%). The most common symptoms were fever (95.1%), cough (78%), myalgia (51.2%) and sore throat (39%). Hypotension (systolic blood pressure<90mmHg) was detected in 6 (14.6%) patients. 

**Table 1:** Clinical and laboratory characteristics of patients with ILI and patients with confirmed novel H1N1flu infection

** Variable **	**Patients with ILI**	**Patients with confirmed Novel H1N1 flu infection**	**P-Value**
**Clinical future: **			
Fever	138 (93.9)	39 (95.1)	p=0.231
Cough	111 (75.5)	32 (78)	p=0.903
Headache	88 (59.9)	24 (58.5)	p= 0.910
Myalgia	87 (59.2)	21 (51.2)	p= 0.289
Sore throat	66 (44.9)	16 (39)	p= 0.495
Coryza	27 (18.4)	10 (24.4)	p=0.281
Vomiting	17 (11.6)	10 (24.4)	p= 0.213
Chest pain	17 (11.6)	7 (17.1)	p= 0.514
Abdominal pain	11 (7.5)	1 (2.4)	p=0.137
Diarrhea	11 (7.5)	3 (7.3)	p= 0.921
Arthralgia	4 (2.7)	2 (4.9)	p=0.336
Hemoptesia	2 (1.4)	1 (2.4)	p= 0.499
Conjunctivitis	2 (1.4)	1 (2.4)	p=0.86
Pletoreha	2 (1.4)	-	-
**Underlying condition:**			
Asthma	18 (12.2)	4 (9.8)	p=0.524
Hypertension	9 (6.1)	2 (4.9)	p=0.231
Diabetes mellitus	12 (8.2)	3 (7.3)	p=0.341
heart failure	3 (2)	2 (4.9)	p= 0.838
BMI≥30	12 (8.2)	2 (4.9)	p= 0.341
Corticosteroid	5 (3.4)	2 (4.9)	p= 0.264
Transplantation	2 (1.4)	1 (2.4)	p=0.499
Chemotherapy	2 (1.4)	1 (2.4)	p=0.113
Smoking	2 (1.4)	2 (4.9)	p=0.504
Pregnancy (% of females)	25 (17)	6 (14.6)	p= 0.709
**Laboratory finding**			
WBC (mean, range)	6497±3270(1500-19200)	7349±3776(600-27000)	p=0.071
Neutrophil	72.1±12.9 (45-93)	72.9±14.5 (35-97)	p=0.723
Lymphocyte	25.9±12.7 (7-55)	23.7±13.8 (2.9-63)	p=090
Hb (mean, range)	12.7±1.9 (7.7-16.5)	12.4±1.8 (6.3-16.5)	p=0.407
PLT (mean, range)	200170±58179 (131000-356000)	198593±68.42 (37600-198000)	p= 0.882
AST (mean, range)	39.3±29.4 (13-171)	38.1±27.2 (10-171)	p=0.452
ALT (mean, range)	35.9±27.5 (10-152)	36±23.9 (7-152	p= 0.583
Serum Cr (mean, range)	0.81±0.3 (0.4-2.3)	0.77±0.33 (0.1-4)	p=0.811
ESR (mean, range)	16.8±15.8 (2-48)	21.6±20.9 (0-107)	p= 0.291

Twenty (48.8%) had one or more underlying medical conditions (including 4 patients with asthma, 3 with diabetes mellitus, 2 with hypertension, 2 with heart failure, 2 with obesity (BMI≥30), 2 with history of corticosteroid therapy and one case with kidney transplantation along immunosuppressive therapy. 

Six pregnant patients were presented as well (14.6% of female cases). Complete blood count was obtained in all the patients, 11 (26.8%) had leuckopenia and only 3 (7.3%) had leukocytosis. Relative lymphopenia (≤21% of white blood cells) was reported in 15 (36.6%). Due to pregnancy, 5 patients did not have a CXR and in 7 cases out of 36 remaining cases, CXR was abnormal. Thrombocytopenia (≤150 000 platelets/mm3) was present in 6 of the 41 (14.6%). The elevation of the liver enzymes (AST, ALT) was seen in 14 of 41 (34.1%). Serum creatinine increased in 2 patients. Serious complications evolved in patients including viral pneumonia in 5 cases (12.2%), secondary bacterial pneumonia in 2 (4.8%), acute pericarditis plus abortion in a pregnant case and aseptic meningitis in one patient. The case of aseptic meningitis was a 30 year old man with severe headache and high fever accompanying chills, generalized myalgia and arthralgia. His CSF analysis was consistent with aseptic meningitis. 

Acute pericarditis was presented in a 25 year old pregnant woman (G_2_P_1_LC_1_) who was transferred to the ICU for mechanical ventilation but had vaginal bleeding which progressed to spontaneous abortion. Antiviral agent (TAMIFLU®) was administered for 36 (87.8%) with usual dosage of 75 mg /BID for 5 days, although 4 patients (include 3 cases with pneumonia and 1 with acute pericarditis) received this drug 150mg/BID. Corticosteroid was prescribed for 10 (24.4%) cases. Four (9.6%) patients were required for mechanical ventilation and ICU care and finally 1 patient unfortunately expired because of severe pneumonia and ischemic heart disease.

## Discussion

In this study, the demographic, clinical and laboratory features of patients with ILI hospitalized in the North of Iran from September 2009 to January 2010 have been reported. The characteristics of these persons with confirmed H1N1 virus infection (41 patients) were compared to the total cases with ILI. These characteristics were overall similar (table 1), although the mean time of patients with obtaining sample for RT-PCR was significantly lower in confirmed H1N1 virus infection to the total cases with ILI. (0.93±0.47 and 4.2±3.8 days, respectively) (p=0.017). Consequently, we concluded that the negative results of RT-PCR in many patients may be due to the delay in sample collection on the other hand, while one study illustrated that among the patients with detectable H1N1 viral RNA in bronchoscopic samples, 19% had negative upper respiratory tract samples (6). 

Accordingly, the negative results in single respiratory specimens do not rule out the 2009 H1N1 virus infection, and the repeated collection of multiple respiratory specimen types is recommended while clinical suspicion is high (7). Approximately 70% of patients with ILI and 80% of confirmed novel H1N1 virus infection were in the age group of 15-45 years. Hence, although this infection involved persons of all age groups, it mostly affected the adolescents and young adults, This finding which is comparative with the result of most current studies (8-10) can presumably be due to the exposure of older persons to antigenically related influenza viruses earlier in life, resulting in the development of cross-protective antibodies (11, 12). 

Thirty nine (95.1%) of confirmed patients had a combination of fever and sore throat or cough, thus in an appropriate epidemiological setting, these symptoms can strongly suggest pandemic influenza. Relative lymphopenia was seen in 36.6% of patients with confirmed novel H1N1 flu infection and 35.2% of patients with ILI. Some studies have stated that relative lymphopenia is a laboratory marker for the early diagnosis of seasonal and pandemic influenza (13). Our findings demonstrated that although relative lymphopenia may be seen in some of these patients, there is no sufficient sensitivity and specificity for distinguishing influenza from the other ILI syndromes. Furthermore, the elevation of liver enzymes was observed in nearly third portion of patients (confirmed or suspected pandemic flu infection). A total of 14.6% of confirmed female cases were pregnant and one of them evolved to have acute pericarditis, vaginal bleeding and abortion. 

In addition, she required intensive care and mechanical ventilation. Influenza in pregnancy is associated with poor outcome in both interpandemic influenza and previous pandemic seasons (14). Consequently, vaccination as the most effective preventive measurement must be certainly considered in these high risk persons. In addition, when epidemic or pandemic influenza virus is circulating in a community, every pregnant patient presenter with ILI must undergo diagnostic and therapeutic measures as soon as possible. Currently, the first wave of pandemic flu has ended and we are waiting for the next possible wave (according to the Northern Hemisphere cold season), so understanding the primary clinical manifestations of disease, risk factors such as pregnancy and other characteristics can help to the earlier detection of infection, timely treatment of patients and proper preventive measurements such as hand washing and household contact avoidance.

## Notes

**Funding: **This project was a grant given by the Vice- Chancellery for Research of Mazandaran University of Medical Sciences.

## References

[1] Zimmer SM, Burke DS (2009). Historical Perspective: Emergence of Influenza A (H1N1) Viruses. N Engl J Med.

[2] Jain S, Kamimoto L, Bramley AM (2009). Hospitalized Patients with 2009 H1N1 Influenza in the United States, April-June 2009. N Engl J Med.

[3] Nickel CH, Stephan FP, Dangel M (2009). First wave of the influenza A/H1N1V pandemic in Switzerland. Swiss Med Wkly.

[4] WHO recommendations for the post-pandemic period. http://www.who.int/csrdisease/swinflu/notes/briefing20100810/en/index.Html.

[5] Interim Guidance on Infection Control measures for 2009 H1N1 influenza in Healthcare settings, including protection of Healthcare personnel. http://www.cdc.gov/h1n1flu/guidelines-infection-control.html.

[6] Blyth CC, Iredell JR, Dwyer DE (2009). Rapid test sensitivity for novel swine-origin influenza A (H1N1) virus in humans. N Engl J Med.

[7] (2009). Bautista E, Chotpitayasunondh T, Gao Z, et al. Clinical Aspects of Pandemic.

[8] Novel Swine-origin influenza A (H1N1) virus investigation team, Dawood FS, Jain S, Finelli L (2009). Emergence of a Novel Swine-Origin Influenza A (H1N1) Virus in Humans. N Engl J Med.

[9] Reed C, Angulo FJ, Swerdlow DL (2009). Estimates of the prevalence of pandemic (H1N1) 2009, United States, April-July 2009. Emerg Infect Dis.

[10] Donaldson LJ, Rutter PD, Ellis BM (2009). Mortality from pandemic A/H1N1 2009 influenza in England: public health surveillance study. BMJ.

[11] Itoh Y, Shinya K, Kiso M (2009). In vitro and in vivo characterization of new swine origin H1N1 influenza viruses. Nature.

[12] Miller E, Hoschler K, Hardelid P (2010). Incidence of 2009 pandemic influenza A H1N1 infection in England: a cross-sectional serological study. Lancet.

[13] Cunha B A, Pherez FM, Schoch P (2009). Diagnostic importance of relative lymphopenia as a marker of swine influenza (H1N1) in adults. Clin infect dis.

[14] Rasmussen SA, Jamieson DJ, Bresee JS (2008). Pandemic influenza and pregnant women. Emerg Infect Dis.

